# The utility of ^18^F-FDG PET/CT in assessing bone marrow involvement and prognosis in newly diagnosed diffuse large B-cell lymphoma

**DOI:** 10.2478/raon-2025-0062

**Published:** 2025-12-16

**Authors:** Chunyan Yang, Hong Liu, Furui Duan, Ximei Wang, Ping Li, Dalong Wang

**Affiliations:** 1 Department of PET/CT, The Second Affiliated Hospital of Harbin Medical University, Harbin, China; 2 Department of Nuclear Medicine and Radiation Oncology, Zibo Central Hospital, Shandong Province, China

**Keywords:** PET/CT, DLBCL, bone marrow biopsy, bone marrow involvement

## Abstract

**Background:**

The presence of bone marrow involvement (BMI) in patients with diffuse large B-cell lymphoma (DLBCL) has a significant impact on treatment plans and prognosis, but clinical diagnosis is difficult. The purpose of this study was to evaluate the utility of PET/CT in the assessment of BMI and prognosis in newly diagnosed DLBCL.

**Patients and methods:**

This retrospective study included 57 eligible DLBCL patients who underwent bone marrow biopsy (BMB) and PET/CT prior to any treatment initiation. Increased FDG uptake in the bone marrow on PET/CT scans was indicative of BMI positivity, with such instances not attributable to benign findings. If BMB yielded positive results, or if the marrow uptake resolved concurrently with other lymphoma lesions during PET/CT monitoring, the diagnosis of BMI was established. The evaluation of bone marrow status via PET/CT involved both visual analysis and a quantitative index, specifically the ratio of maximum standardized uptake values of bone marrow to liver (BLR). Factors associated with 2-year progression-free survival (PFS) was analyzed utilizing the Cox proportional hazards regression model.

**Results:**

34 patients were diagnosed with BMI. PET/CT demonstrated superior accuracy (93.0% *vs*. 75.4%) and sensitivity (94.1% *vs*. 58.8%) compared to BMB. During the follow-up period, 15 patients experienced disease progression. Survival analysis identified Eastern Cooperative Oncology Group performance status (ECOG PS), BLR, and PET/CT bone marrow status as the sole independent predictors of PFS (p = 0.010, 0.002, and 0.015, respectively).

**Conclusions:**

PET/CT played an important role in evaluating BMI and predicting PFS in newly diagnosed DLBCL.

## Introduction

Diffuse large B-cell lymphoma (DLBCL) represents a prevalent and aggressive form of lymphoma, constituting approximately 30% of all non-Hodgkin lymphoma cases.^1^ Bone marrow involvement (BMI) serves as a critical prognostic indicator for lymphoma patients, significantly impacting disease staging and prognosis. Accurate determination of BMI is therefore paramount. Traditionally, bone marrow biopsy (BMB) has been regarded as the gold standard for assessing BMI in DLBCL due to its ability to evaluate the bone marrow (BM) status of lymphoma patients. However, the advent and advancement of nuclear imaging technology have posed a challenge to its longstanding position in clinical practice. In previous literature reports, its conventional performance has been controversial. BMB, as an invasive procedure, carries the potential for patient anxiety and the risk of bleeding. Moreover, it is prone to yield false negative outcomes, particularly in cases where the true lesion is missed due to biopsy site limitations, notably with respect to distant BM lesions from the iliac spine.^2,3^ Furthermore, certain studies propose that routine BMB may lack therapeutic relevance.^4^

^18^F-FDG positron emission tomography (PET)/computed tomography (CT) is capable of accurately depicting the glucose metabolism status of tissues or lesions through the utilization of its glucose analogues, offering vital physiological and pathological insights for clinical utility. The heightened metabolic activity of malignant tumor cells results in significant FDG uptake, rendering PET/CT particularly advantageous for diagnostic purposes, disease staging, treatment response assessment, and surveillance of lymphoma recurrence. Whole-body PET/CT imaging enables comprehensive visualization of tumor location, morphology, size, and extent, aligning with recommendations from the European Society for Medical Oncology guidelines as the preferred modality for evaluating systemic disease dissemination in DLBCL patients.^5^ Highlights that, in comparison to BMB, PET/CT imaging allows for the assessment of BM status throughout the entire body rather than at a single anatomical site. In a recent study by Doma *et al*. found that the sensitivity and accuracy of PET/CT in diagnosing BMI were significantly higher than BMB, at 88.4% *vs*. 41.9% and 96.5% *vs*. 61.5%, respectively.^6^ They advocated for PET/CT as a viable alternative to BMB for evaluating BMI in patients with DLBCL. Nonetheless, the precise role of BM FDG uptake in DLBCL patients remains a topic of ongoing discussion.

In this study, we aim to assess the correlation and prognostic significance of PET/CT and BMB in BMI among newly diagnosed DLBCL patients, providing clinical practitioners with diagnostic insights and therapeutic considerations to aid in treatment decision-making processes.

## Patients and methods

### Patients

A retrospective analysis was conducted on DLBCL patients who underwent PET/CT scans between 2017 and 2024. These patients had not undergone radiotherapy, chemotherapy, hematopoietic factor therapy, or had a previous history of other malignancies prior to the PET/CT scans. Patient confidentiality was maintained in compliance with national regulations. The study was approved by the Ethics Committee of the Second Affiliated Hospital of Harbin Medical University (Approval No. YJSKY2023-327, 2023-09-04).

Clinical data was sourced from institutional information systems. A review was conducted on the following clinical parameters: age, sex, Eastern Cooperative Oncology Group performance status (ECOG PS), B-symptoms, clinical manifestations (pain, nosebleeds, loss of appetite, bloating, among others), and extranodal involvement. The baseline laboratory findings obtained within a 2-week window before and after PET/CT imaging encompassed serum lactate dehydrogenase (LDH), lymphocyte-to-monocyte ratio (LMR > 3), neutrophil-to-lymphocyte ratio (NLR > 2.5)^7^, leukopenia (< 4×10^9^/L), anemia (hemoglobin < 120g/L), and thrombocytopenia (platelet counts < 100×10^9^/L). The International Prognostic Index (IPI) score was based on these data. Following PET/CT scans, all patients underwent first-line therapy. The study cohort was derived according to the process detailed in Supplementary Figure 1.

### Acquisition of PET/CT images

Imaging was performed using a PET/CT system (Biograph64 mCT, Siemens Healthcare, Berlin, Germany) with a full-ring PET scanner. Patients fasted for 6 hours and maintained blood glucose levels below 11 mmol/L before the procedure. After FDG injection (0.11 mCi/kg), patients rested supine for 60 minutes. CT was performed with a 120 kV tube voltage and modulated tube current (CARE Dose). PET acquisition was conducted at 1.6 mm/s, and images were reconstructed with the TrueD algorithm for attenuation correction using CT data. The PET matrix was 200×200 (voxel size: 4.07×4.07×3 mm), and the CT matrix was 512×512 (voxel size: 0.78×0.78×1 mm), with PET images reconstructed using the TrueX + TOF method.

### Evaluation of PET/CT images and analysis of FDG uptake

**FIGURE 1.**
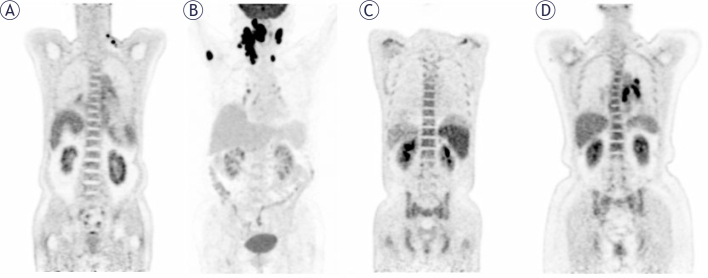
The initial PET maximum-intensity projection images, and lymph nodes pathology confirmed DLBCL with normal **(A)**, focal increased in the right humerus **(B)**, diffuse increased **(C, D)**.

Image interpretation was conducted through visual and semi-quantitative analysis, utilizing glucose activity in the normal liver as a reference to assess the presence of BMI. In cases where BM lesions were identified, they were classified as focal lesions (demonstrating elevated FDG uptake in one or more localized regions) or diffuse lesions (exhibiting a uniform increase in FDG uptake across the entire marrow space). These lesions, with or without associated bone destruction, were distinguished from benign findings based on standard CT images or medical history (*e.g*., fractures). In such cases, BM positivity on PET/CT was ascertained. The maximum standardized uptake value (SUVmax) of the liver was measured in a 2-cm region of interest in the right lobe, while the highest SUVmax for BM was assessed at lumbar vertebrae 1-5 in cases of diffuse uptake. The BM-to-liver SUVmax ratio (BLR) was calculated to investigate survival outcomes. Two experienced nuclear medicine specialists, each possessing more than 5 years of professional experience, independently interpreted the images, with discrepancies resolved by consensus (Figure 1).

### Bone marrow biopsy (BMB) and pathological analysis

All patients underwent unilateral iliac BM core needle biopsy and aspiration without imaging guidance. BMB samples were collected within one month before or after the PET/CT imaging and evaluated by a senior hematologist.

### Definition of the final diagnosis of bone marrow involvement (BMI)

If BM uptake paralleled the uptake activity of other lymphoma lesions during follow-up with PET/CT or was confirmed by BMB, the diagnosis of BMI was confirmed.^8,9^

### Statistical analysis

Receiver operating characteristic (ROC) curve analysis was employed to establish the optimal BLR threshold for survival analysis. Progression-free survival (PFS) was defined as the duration from the definitive diagnosis to the first occurrence of disease progression or relapse, all-cause mortality, or the most recent follow-up. The Shapiro-Wilk test was utilized for assessing the distribution of continuous variables in the baseline data. Continuous variables were expressed as median and interquartile range. Categorical data were presented as patient counts and percentages. Disparities in variables between the two groups were assessed using logistic regression analysis and the chi-square test. Comparison of SUVmax and BLR was conducted using the Kruskal-Wallis test and Dunn’s post-hoc test. Cox proportional hazards regression models were utilized to analyze the impact of factors such as IPI and related components, BLR, and BM status on survival outcomes. Survival analysis was performed using Kaplan-Meier curves and log-rank tests. Statistically significant variables (p < 0.05) from the univariate analysis were entered into the multivariate Cox regression. The resulting independent prognostic factors were incorporated to construct a prognostic nomogram. The model was validated internally using bootstrap resampling with 1,000 iterations. The discriminative ability of the nomogram was quantified by the concordance index (C-index), and its accuracy was visually assessed with a calibration curve comparing predicted probabilities against observed frequencies. Statistical analyses were performed using IBM SPSS 27 and R 4.3.3 software.

## Results

### Clinical characteristics

In this study, a total of 57 DLBCL cases were analyzed in Table 1. The cohort showed 27 males (47.4%) and 30 females (52.6%), with a median age of 61 years old (range: 28–81). Among 57 patients, 30 (52.6%) were aged 60 years or older.

**TABLE 1.** Baseline characteristics at diagnosis

Characteristic	PET/CT		*p* -value	BMB		*p*-value
	(-) (n = 23)	(+) (n = 34)		(-) (n = 37)	(+) (n = 20)	
Male, no. (%)	11 (47.83)	16 (47.06)	0.955	17 (45.95)	10 (50.00)	0.770
Leukopenia, no. (%)	2 (8.70)	6 (17.65)	0.571	2 (5.41)	6 (30.00)	0.031
Anaemia, no. (%)	4 (17.39)	15 (44.12)	0.036	7 (18.92)	12 (60.00)	0.002
Thrombocytopenia, no. (%)	2 (8.70)	8 (23.53)	0.276	3 (8.11)	7 (35.00)	0.029
LMR > 3, no. (%)	15 (65.22)	20 (58.82)	0.627	26 (70.27)	9 (45.00)	0.061
NLR > 2.5, no. (%)	10 (43.5)	21 (61.8)	0.174	19 (51.40)	12 (60.00)	0.532
Age > 60 years, no. (%)	10 (43.48)	20 (58.82)	0.255	19 (51.35)	11 (55.00)	0.792
Stage III/IV, no. (%)	19 (82.61)	29 (85.29)	1	28 (75.68)	20 (100)	0.043
ECOG PS ≥ 2, no. (%)	8 (34.78)	14 (41.18)	0.627	11 (29.73)	11 (55.00)	0.061
Clinical systemic symptoms, no. (%)	21 (91.30)	25 (73.53)	0.185	31 (83.78)	15 (75.00)	0.652
Biological systemic symptoms, no. (%)	11 (47.83)	20 (58.82)	0.413	18 (48.65)	13 (65.00)	0.237
LDH (+), no. (%)	8 (34.78)	18 (52.94)	0.177	15 (40.54)	11 (55.00)	0.296
IPI score > 2, no. (%)	8 (34.78)	18 (52.94)	0.177	12 (32.43)	14 (70.00)	0.007
Extranodal sites ≥ 2	6 (26.09)	13 (38.24)	0.340	8 (21.62)	11 (55.00)	0.011
BMB (+), no. (%)	2 (8.70)	18 (52.94)	< 0.001	-	-	-
PET (+), no. (%)	-	-	-	16 (43.24)	18 (90.00)	< 0.001
High level of BLR, no. (%)	0	27 (79.41)	< 0.001	10 (27.03)	17 (85.00)	< 0.001
BM SUVmax, median (P25, P75)	2.48 (2.12,3.02)	4.74 (3.42,9.43)	0.002	2.78 (2.28,3.55)	6.39 (4.47,11.73)	0.003

1 BLR = the ratio of the maximum standardized uptake values of bone marrow-to-liver; BMB = bone marrow biopsy; BM SUVmax = maximum standardized uptake value of bone marrow; ECOG PS = Eastern Cooperative Oncology Group physical status score; IPI = international prognostic index; LDH = lactate dehydrogenase; LMR = lymphocyte-to-monocyte ratio; NLR = neutrophil-to-lymphocyte ratio

The baseline characteristics of the patients defined by the two diagnostic methods – (1) the PET/CT-based cohort and (2) the BMB-based cohort – were compared. There were no significant differences between the two groups in terms of sex, age, LMR > 3, NLR > 2.5, clinical symptoms, B-symptoms, LDH (+), ECOG PS ≥ 2, extranodal sites ≥ 2, or IPI score > 2. However, there were statistical differences in the anemia, BM SUVmax, and BLR levels (all p < 0.05).

### PET/CT and BMB diagnostic performance

Out of the 34 patients detected as BMI positive by PET/CT, 32 were confirmed as true positive cases (20 confirmed via BMB, 12 through follow-up). Among the 23 patients identified as BMI negative by PET/CT, 2 were verified as false negative by BMB, while the remaining 21 were confirmed as negative by BMB or follow-up assessments (Table 2).

**TABLE 2.** Comparison of bone marrow biopsy (BMB) and PET/CT results with bone marrow involvement (BMI)

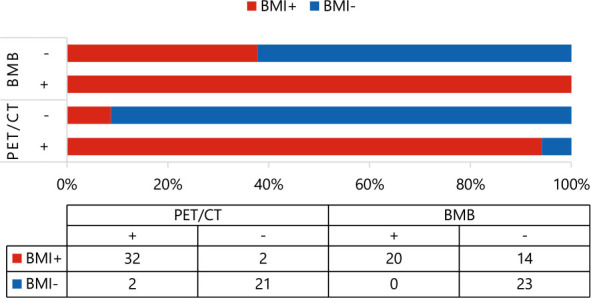

We identified 34 patients (59.6%) with BMI and 23 (40.4%) without BMI. Using the final clinical diagnosis as the reference standard, PET/CT correctly identified 32 (56.1%) true positive cases, while BMB correctly identified 20 (35.1%) true positive cases. The diagnostic results from PET/CT and BMB were consistent in 39 cases. Specifically, 18 (31.6%) demonstrated concordant positive findings on both BMB and PET/CT tests, while 21 (36.8%) displayed negative outcomes for BMB and PET/CT. However, there were 18 (31.6%) cases where the diagnostic results between PET/CT and BMB were inconsistent, including 16 (28.1%) that tested positive for PET/CT yet negative for BMB, and 2 (3.5%) that tested negative for PET/CT but positive for BMB (Table 3).

**TABLE 3.** Diagnostic performance in all diffuse large B-cell lymphoma (DLBCL) patients

		PET/CT		Total
		(+)	(-)	
**BMB**	(+)	18	2	20
	(-)	16	21	37
**Total**		34	23	57

1 BMB = bone marrow biopsy

Table 4 compares the diagnostic performance of PET/CT and BMB against the pre-defined BMI. PET/CT demonstrated superior diagnostic efficacy, with an accuracy of 93.0% and a sensitivity of 94.1%. Its specificity, positive predictive value (PPV), and negative predictive value (NPV) were 91.3%, 94.1%, and 91.3%, respectively. The high Youden index (0.854) and excellent agreement with the reference standard (kappa = 0.854) further confirm its robustness.

**TABLE 4.** Comparison of bone marrow biopsy (BMB) and PET/CT to detect bone marrow involvement (BMI)

	Accuracy (%)	Sensitivity (%)	Specificity (%)	PPV (%)	NPV (%)	YI	kappa	AUC
PET/CT (BMB as standard)	68.4 (95 CI,76.9–100)	56.8 (95 CI, 40.8–72.7)	52.9 (95 CI, 36.2–69.7)	52.9 (95 CI, 36.2–69.7)	91.3 (95 CI, 79.8–100)	0.468	0.403	0.734
PET/CT (BMI as standard)	93.0 (95 CI, 92.8–93.2)	94.1 (95 CI, 86.2–100)	91.3 (95 CI, 79.8–100)	94.1 (95 CI, 86.2–100)	91.3 (95 CI, 79.8–100)	0.854	0.854	0.927
BMB (BMI as standard)	75.4 (95 CI, 74.8–76.1)	58.8 (95 CI,42.3–75.4)	100 (95 CI,100–100)	100 (95 CI,100–100)	62.2 (95 CI,46.5–77.8)	0.588	0.536	0.794

1 AUC = area under ROC curve; CI = confidence interval; NPV = negative predictive value; PPV = positive predictive value; YI = Youden index

### PET/CT characteristics

Utilizing the optimal cut-off value of BLR = 1.340 (AUC = 0.755, p = 0.004), patients were stratified into high- and low-BLR groups. All 27 patients (100%) in the high-BLR group were interpreted as positive by visual PET/CT assessment. In contrast, only 7 of the 30 patients (23.3%) in the low-BLR group were PET/CT-positive. The agreement between the quantitative (BLR-based) and visual PET/CT assessments was excellent (p < 0.001, kappa = 0.757).

**FIGURE 2.**
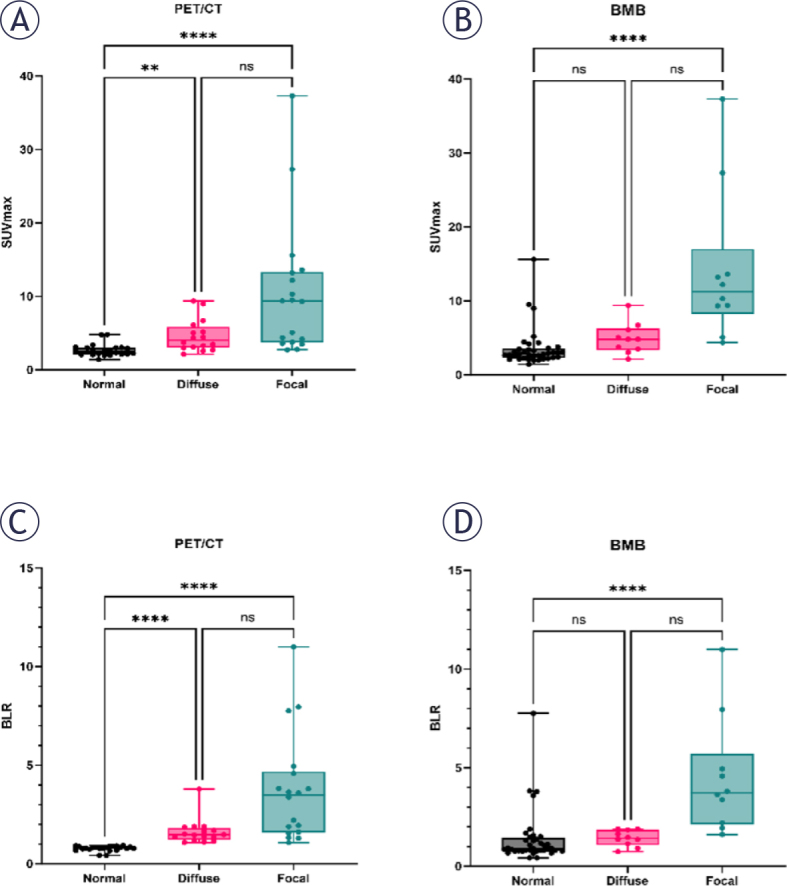
Distribution of uptake according to bone marrow biopsy (BMB) and PET/CT. **, p = 0.006; ****, p < 0.0001; BLR = the ratio of the maximum standardized uptake values of bone marrow-to-liver; ns = statistically nonsignificant; SUVmax = maximum standardized uptake value of bone marrow

Among all patients, the median SUVmax of 23 patients with normal BM uptake was 2.48 (2.12, 3.02), and the BLR was 0.80 (0.75, 0.87). In contrast, the median SUVmax of 18 patients with focal BM uptake was 9.36 (3.73, 13.30; p < 0.001), BLR was 3.49 (1.60, 4.67; p < 0.001), and the median SUVmax of 16 patients with diffuse BM uptake was 4.04 (3.04, 5.86; p = 0.006), BLR was 1.48 (1.23, 1.81; p < 0.001). The difference in SUVmax and BLR between focal and diffuse uptake groups was not statistically significant (Figure 2A and C). The median SUVmax and BLR of 34 patients with increased BM uptake were 1.77 (1.37, 3.68) and 4.74 (3.42, 9.43), respectively, with statistical differences (both p < 0.001). Using BMB as the standard for analysis, the median SUVmax and BLR of 37 cases of BMB (-) were 2.78 (2.28, 3.55) and 0.79 (0.89, 1.44), while 20 cases of BMB (+) had higher median SUVmax and BLR, which were 6.39 (4.47, 11.73; p < 0.001) and 1.89 (1.40, 3.78; p < 0.001), respectively. The median SUVmax of focal and diffuse BM uptake were 11.25 (8.25, 17.03) and 4.80 (3.35, 6.24), respectively, and the median BLR were 3.72 (2.15, 5.70) and 1.42 (1.09, 1.86), respectively (Figure 2B and D). The 18 patients who were positive on both PET/CT and BMB exhibited significantly elevated median SUVmax and BLR compared to the rest of the cohort: SUVmax 8.00 (4.20-12.45) *vs*. 2.92 (2.32-3.78), and BLR 1.92 (1.57-4.00) *vs*. 0.89 (0.78-1.39); all comparisons were statistically significant (p < 0.001).

### PET/CT and BMB findings on staging

According to the Ann Arbor stage, PET/CT and BMB were independently assessed. PET/CT classified 46 patients (80.7%) as stage IV, while BMB classified 44 patients (77.2%) as stage IV. The final clinical diagnosis, which integrated all available data, led to the downstaging of two patients: one from stage to I stage IV, and another from stage III to stage IV. This demonstrates that PET/CT plays a crucial role in preventing understaging, thereby ensuring more accurate disease staging.

### Survival analysis

**FIGURE 3.**
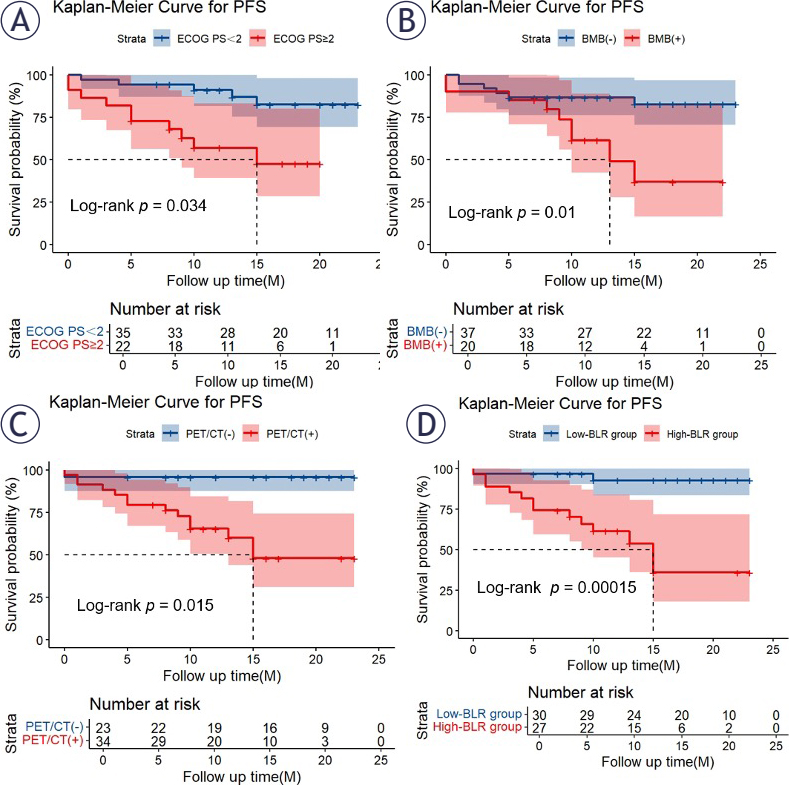
Kaplan-Meier curves for progression-free survival (PFS) in total study patients according to Eastern Cooperative Oncology Group performance status (ECOG PS) **(A)**, bone marrow biopsy (BMB) **(B)**, PET/CT **(C)** and the ratio of the maximum standardized uptake values of bone marrow-to-liver (BLR) groups **(D)**

All 57 patients were included in the survival analysis. The median survival time was not reached at the time of analysis, with 15 patients experiencing disease progression or death. 34 patients were BMI (+), with a median PFS of 15 months and the 2-year PFS rate 47.9% ± 10.7%. In visual analysis, both a positive BMB (p = 0.016, Figure 3A) and a positive PET/CT finding (p = 0.012, Figure 3B) were significantly associated with shorter PFS; In quantitative analysis, high BLR levels (cut-off value, 1.340) were correlated with shorter PFS (p < 0.001, Figure 3C). The 2-year PFS rates were 92.6% ± 5.0% for the low-BLR group and 35.8% ± 12.7% for the high-BLR group.

Variables found to be significant in the univariate Cox analysis for PFS are listed in Table 5. Multivariate analysis confirmed that high BLR levels and an ECOG PS ≥ 2 were independent risk factors for shorter PFS (Table 5). Kaplan-Meier survival plots visually corroborated the poor prognosis associated with a high BLR and an ECOG PS ≥ 2 (Figure 3A and D).

**TABLE 5.** Univariate and multivariate Cox analysis of risk factors for progression-free survival (PFS) in 57 patients with diffuse large B-cell lymphoma (DLBCL)

Characteristics	Univariable		Multivariable	
	HR (95%CI)	*p*-value	HR (95%CI)	*p*-value
Male	0.619 (0.220-1.747)	0.365		
Leukopenia	0.370 (0.049-2.821)	0.338		
Anaemia	1.880 (0.680-5.199)	0.224		
Thrombocytopenia	0.772 (0.174-3.429)	0.734		
LMR > 3	0.251 (0.085-0.737)	0.012		
NLR > 2.5	2.763 (0.877-8.708)	0.083		
Age<60 years	1.270 (0.458-3.521)	0.646		
Clinical systemic symptoms	1.500 (0.338-6.656)	0.594		
Biological systemic symptoms	0.636 (0.217-1.866)	0.410		
Stage III or IV	1.268 (0.286-5.624)	0.755		
ECOG PS ≥ 2	4.368 (1.479-12.896)	0.008	4.286 (1.422-12.919)	0.010
LDH (+)	3.856 (1.227-12.122)	0.021		
Extranodal sites ≥ 2	3.638 (1.292-10.245)	0.014		
IPI score<2	3.110 (1.055-9.163)	0.040		
BMB (+)	3.663 (1.267-10.415)	0.016		
PET (+)	12.850 (1.678-98.407)	0.014		
High level of BLR	10.527 (2.340-47.364)	0.002	10.518 (2.305-47.986)	0.002
BM SUVmax	1.021 (0.954-1.093)	0.543		

1 BMB = bone marrow biopsy; BLR = the ratio of the maximum standardized uptake values of bone marrow-to-liver; ECOG PS = Eastern Cooperative Oncology Group performance status; CI = confidence interval; ECOG PS = Eastern Cooperative Oncology Group physical status score; IPI = international prognostic index; LDH = lactate dehydrogenase; LMR = lymphocyte-to-monocyte ratio; NLR = neutrophil-to-lymphocyte ratio

Based on the results of the multivariate analysis, we constructed a nomogram for predicting PFS (Supplementary Figure 2). The nomogram demonstrated good predictive accuracy for PFS, with a C-index of 0.812. The calibration curve for PFS survival probabilities showed strong agreement between the actual observed probabilities and those predicted by the nomogram (Supplementary Figure 3).

## Discussion

In our retrospective study, we observed that PET/CT scans exhibited superior diagnostic accuracy compared to BMB, which was consistent with findings from various other studies reporting the heightened sensitivity of PET/CT over BMB.^6,8–12^ Reviews and meta-analyses conducted further supported these observations, highlighting the superior performance of PET/CT in assessing BMI in DLBCL patients, detecting additional marrow infiltration missed by BMB, and affirming the higher diagnostic value of FDG PET/CT results over BMB.^13–15^

Numerous studies and reviews have highlighted the notable sensitivity of PET/CT in evaluating BM status in DLBCL patients.^6,8–11,16,17^ For newly diagnosed DLBCL patients, the detection of negative BM uptake on PET/CT imaging may obviate the need for BMB, unless there are apprehensions regarding potential oversight of low-grade lymphoma involvement by PET/CT. Nevertheless, it has yet to be established that PET/CT can serve as a definitive substitute for BMB in clinical practice. In our study, a total of 34 patients were assessed as BMI through combined PET/CT monitoring and follow-up, a proportion higher than that reported in previous studies (11–50%).^8,9,18,19^ This higher detection rate may be attributed to the predominance of advanced-stage patients (91.2%) in our cohort, wherein PET/CT imaging and follow-up monitoring could more effectively detect early metastatic lymphoma infiltration in the BM. Furthermore, following the detection of BMI via PET/CT, 2 patients (3.5%) in our cohort were reclassified with advanced stage, aligning with findings from previous research. Berthet *et al*. and Pelosi *et al*. reported upstaging rates of 10% and 21%, respectively, through PET/CT evaluations.^12,20^ PET/CT demonstrates enhanced accuracy in staging and BMI detection. Assessing infiltration in cases with partially diffuse BM uptake can be challenging, as it may result from inflammatory or proliferative conditions. In our study, 8 out of 10 cases displaying diffusely increased BM uptake were confirmed as DLBCL via BMB, mirroring the heightened BMI incidence in the diffuse BM uptake pattern investigated through many studies.^11,12,19,21,22^ There were 2 cases of diffuse FDG metabolic increase with BMB (-), which may be caused by various factors, such as active marrow hyperplasia, inflammation, or infection, rather than malignant infiltration.^23,24^ In this study, the median SUVmax of BM in patients with diffuse BM FDG uptake was higher than normal (4.04 *vs*. 2.48, p = 0.006). A study conducted by Lim *et al*. reported a notable disparity (p < 0.001) in the visual evaluation of BM uptake between 64 patients diagnosed with DLBCL and the remaining 448 patients showing no elevation in BM uptake.^24^ Xiao-Xue *et al*. also reached a similar conclusion, and their study showed that the median values of SUVmax in the patients detected to have BMB (+) were significantly higher than patients with BMB (-) among subgroups of aggressive B-cell lymphoma, marginal zone lymphoma, T cell non-Hodgkin’s lymphoma (p < 0.05).^25^ Based on previous researches, we believe that the observed BM uptake pattern should be considered an indicator or high-risk factor for DLBCL BM infiltration, given the relatively low rate of false positives in cases of diffuse BM uptake on PET/CT. In this study, two patients with negative PET/CT results were later confirmed to have bone lesions upon histological biopsy. However, due to the diverse morphologies of tumor cells, the specific type of lymphoma could not be definitively identified. This ambiguity may be attributed to either a low tumor burden or smallcell (discordant) BMI, which could potentially exhibit limited FDG avidity, leading to potential oversight by PET/CT imaging.^26,27^ We recommend multidisciplinary review or next-generation sequencing (NGS) to resolve PET/CT-BMB discordance.

In our study, univariate analysis identified LMR > 3, ECOG PS ≥ 2, LDH (+), extranodal sites ≥ 2, IPI > 2, BMB (+), PET/CT (+), and high-BLR group as factors associated with 2-year PFS, while multivariate analysis found high-BLR group and ECOG PS ≥ 2 to be independent predictors. In the study by Lim *et al*., elevated BM FDG uptake was linked to patient prognosis in cases with positive biopsy results (HR = 2.79; p = 0.008), a correlation that aligns with the outcomes of our research.^24^ El Karak *et al*. similarly documented, in a study involving 54 patients with DLBCL prior to treatment, that PET held prognostic significance in BMI, which was closely associated with PFS (HR = 3.81, p = 0.013) and overall survival (OS) (HR = 4.12; p = 0.03), while BMB did not.^16^ Similar finding was reported by Berthet *et al*., who conducted a study comparing PFS and OS at the 2-year mark in 133 patients based on BMI as determined by BMB and PET/CT.^12^ Their research indicated that BM disease detected on PET was an independent prognostic indicator for both PFS and OS, a result consistent with the prognostic value observed for BMI determined by BMB. Interestingly, other researchers reported differing results, noting that BMI identified on PET did not hold prognostic significance, with only BMI identified by BMB serving as an independent predictive factor.^9,21,22,28^ Chen-Liang *et al*. noticed in their study that BMB (-) BMI (+) was independently associated with shorter PFS (HR = 3.6; p = 0.001), rather than PET/CT (-) BMI (+), which contradicts our results.^19^ In addition, as a quantitative indicator of BM uptake, we found that BLR levels were independently associated with PFS in lymphoma patients. Chen *et al*. found PET (+) to be of great significance for predicting PFS and OS.^29^ Interestingly, BLR (cut-off value, 1.50) was significant for PFS and OS (p < 0.001 and p = 0.002), resonating well with our findings. It is noteworthy to highlight that El Azony *et al*., in a study encompassing 135 patients with newly diagnosed DLBCL, concluded that the BLR served as a prognostic factor for recurrence free survival (RFS) and OS in patients with DLBCL (HR = 2.83, p = 0.030 and HR = 2.38, p = 0.041).^30^ Only a small number of studies addressed and supported the idea that BLR was associated with the prognosis of PFS and OS in lymphoma patients, and was even an independent predictor.^13,29,31^

The variations observed in PFS outcomes across studies may be attributed to differences in patient demographics and the duration of follow-up. Distinguishing our study from prior research, we specifically assessed BMI in patients with DLBCL. Currently, there is limited literature on the prognostic value of BLR in B-cell lymphoma, especially in the context of DLBCL. Our research findings provide valuable references for future studies. In addition, different methods between studies may lead to differences in the determination of BM status, as some rely solely on BMB, which may result in false negative results.

We recognize the limitations inherent in our research design. Our study is retrospective and conducted at a single institution with a relatively small cohort, potentially introducing selection and information bias into the data. Additionally, one of the criteria for diagnosing BMI involves the reduction or disappearance of intense focal uptake, a condition that could lead to false positive results. Prospective large-scale studies are needed to further verify these results.

## Conclusions

In summary, our research findings indicate that PET/CT and BMB are complementary in evaluating BMI and predicting prognosis in patients with DLBCL. Additionally, high BLR levels are an independent factor affecting PFS in patients.

## Supplementary Material

Supplementary Material Details

## References

[1] Alaggio R, Amador C, Anagnostopoulos I, Attygalle AD, Araujo IBdO, Berti E (2022). The 5th edition of the World Health Organization classification of haematolymphoid tumours: lymphoid neoplasms. Leukemia.

[2] Teagle AR, Barton H, Charles-Edwards E, Dizdarevic S, Chevassut T. (2017). Use of FDG PET/CT in identification of bone marrow involvement in diffuse large B cell lymphoma and follicular lymphoma: comparison with iliac crest bone marrow biopsy. Acta Radiologica.

[3] Hopkins E, Devenish G, Evans G, Leopold G, Rees J, Parry-Jones N. (2014). Subcutaneous seeding of ‘double hit’ diffuse large B-cell lymphoma from staging bone marrow biopsy. Br J Haematol.

[4] Adams HJA, de Klerk JMH, Fijnheer R, Heggelman BGF, Dubois SV, Nievelstein RAJ (2014). Bone marrow biopsy in diffuse large B-cell lymphoma: useful or redundant test?. Acta Oncol.

[5] Tilly H, Gomes da Silva M, Vitolo U, Jack A, Meignan M, Lopez-Guillermo A (2015). Diffuse large B-cell lymphoma (DLBCL): ESMO Clinical Practice Guidelines for diagnosis, treatment and follow-up. Ann Oncol.

[6] Doma A, Zevnik K, Studen A, Prevodnik VK, Gasljevic G, Novakovic BJ. (2024). Detection performance and prognostic value of initial bone marrow involvement in diffuse large B-cell lymphoma: a single centre 18F-FDG PET/CT and bone marrow biopsy evaluation study. Radiol Oncol.

[7] Chen Y, Xu J, Meng J, Ding M, Guo Y, Fu D (2023). Establishment and evaluation of a nomogram for predicting the survival outcomes of patients with diffuse large B-cell lymphoma based on International Prognostic Index scores and clinical indicators. Ann Transl Med.

[8] Kaddu-Mulindwa D, Altmann B, Held G, Angel S, Stilgenbauer S, Thurner L (2021). FDG PET/CT to detect bone marrow involvement in the initial staging of patients with aggressive non-Hodgkin lymphoma: results from the prospective, multicenter PETAL and OPTIMAL>60 trials. Eur J Nucl Med Mol Imaging.

[9] Khan AB, Barrington SF, Mikhaeel NG, Hunt AA, Cameron L, Morris T (2013). PET-CT staging of DLBCL accurately identifies and provides new insight into the clinical significance of bone marrow involvement. Blood.

[10] Elamir Y, Elazab M, Owis AS, Elsayed HF. (2020). PET/CT and bone marrow biopsy (BMB) in evaluating bone marrow in lymphoma. Egypt J Radiol Nucl Med.

[11] Büyükşimşek M, Kolsuz İ, Yetişir AE, Tohumcuoğlu M, Oğul A, Mirili C (2020). Performance of positron emission tomography-computed tomography and bone marrow biopsy in detecting bone marrow infiltration in lymphoma cases. Turk J Haematol.

[12] Berthet L, Cochet A, Kanoun S, Berriolo-Riedinger A, Humbert O, Toubeau M (2013). In newly diagnosed diffuse large B-cell lymphoma, determination of bone marrow involvement with 18F-FDG PET/CT provides better diagnostic performance and prognostic stratification than does biopsy. J Nucl Med.

[13] Pakos EE, Fotopoulos AD, Ioannidis JP. (2005). 18F-FDG PET for evaluation of bone marrow infltration in staging of lymphoma: a meta-analysis. J Nucl Med.

[14] Li Z, Li C, Chen B, Shi L, Gao F, Wang P (2021). FDG-PET/CT versus bone marrow biopsy in bone marrow involvement in newly diagnosed paediatric lymphoma: a systematic review and meta-analysis. J Orthop Surg Res.

[15] Adams HJA, Kwee TC, de Keizer B, Fijnheer R, de Klerk JMH, Nievelstein RAJ. (2013). FDG PET/CT for the detection of bone marrow involvement in diffuse large B-cell lymphoma: systematic review and meta-analysis. Eur J Nucl Med Mol Imaging.

[16] El Karak F, Bou-Orm IR, Ghosn M, Kattan J, Farhat F, Ibrahim T (2017). PET/CT scanner and bone marrow biopsy in detection of bone marrow involvement in diffuse large B-cell lymphoma. PLoS One.

[17] Al-Sabbagh A, Ibrahim F, Szabados L, Soliman DS, Taha RY, Fernyhough LJ. (2020). The role of integrated positron emission tomography/computed tomography (PET/CT) and bone marrow examination in staging large B-cell lymphoma. Clin Med Insights Oncol.

[18] Mittal BR, Manohar K, Malhotra P, Das R, Kashyap R, Bhattacharya A (2011). Can fluorodeoxyglucose positron emission tomography/computed tomography avoid negative iliac crest biopsies in evaluation of marrow involvement by lymphoma at time of initial staging?. Leuk Lymphoma.

[19] Chen-Liang TH, Martín-Santos T, Jerez A, Rodríguez-García G, Senent L, Martínez-Millán C (2017). Bone marrow biopsy superiority over PET/CT in predicting progression-free survival in a homogeneously-treated cohort of diffuse large B-cell lymphoma. Cancer Med.

[20] Pelosi E, Penna D, Douroukas A, Bellò M, Amati A, Arena V (2011). Bone marrow disease detection with FDG-PET/CT and bone marrow biopsy during the staging of malignant lymphoma: results from a large multicentre study. Q J Nucl Med Mol Imaging.

[21] Hong J, Lee Y, Park Y, Kim SG, Hwang KH, Park SH (2011). Role of FDG-PET/CT in detecting lymphomatous bone marrow involvement in patients with newly diagnosed diffuse large B-cell lymphoma. Ann Hematol.

[22] Adams HJA, Kwee TC, Fijnheer R, Dubois SV, Nievelstein RAJ, de Klerk JMH. (2014). Bone marrow 18F-fluoro-2-deoxy-d-glucose positron emission tomography/computed tomography cannot replace bone marrow biopsy in diffuse large B-cell lymphoma. Am J Hematol.

[23] Zhou M, Chen Y, Liu J, Huang G. (2018). A predicting model of bone marrow malignant infiltration in ^18^F-FDG PET/CT images with increased diffuse bone marrow FDG uptake. J Cancer.

[24] Lim CH, Hyun SH, Cho YS, Choi JY, Lee KH. (2021). Prognostic significance of bone marrow 2-[^18^F]-fluoro-2-deoxy-d-glucose uptake in diffuse large B-cell lymphoma: relation to iliac crest biopsy results. Clin Radiol.

[25] Xiao-Xue W, Xinyue H, Lijun Z. (2020). Whole body FDG-PET/CT for the assessment of bone marrow infiltration in patients with newly diagnosed lymphoma. Med Clin (Barc).

[26] Paone G, Itti E, Haioun C, Gaulard P, Dupuis J, Lin C (2008). Bone marrow involvement in diffuse large B-cell lymphoma: correlation between FDG-PET uptake and type of cellular infiltrate. Eur J Nucl Med Mol Imaging.

[27] Alzahrani M, El-Galaly TC, Hutchings M, Hansen JW, Loft A, Johnsen HE (2016). The value of routine bone marrow biopsy in patients with diffuse large B-cell lymphoma staged with PET/CT: a Danish-Canadian study. Ann Oncol.

[28] Vishnu P, Wingerson A, Lee M, Mandelson MT, Aboulafia DM. (2017). Utility of bone marrow biopsy and aspirate for staging of diffuse large B cell lymphoma in the era of positron emission tomography with 2-deoxy-2-[Fluorine-18]fluoro-deoxyglucose integrated with computed tomography. Clin Lymphoma Myeloma Leuk.

[29] Chen J, Zhao Y. (2024). Pre-treatment [^18^F]FDG PET/CT for assessing bone marrow involvement and prognosis in patients with newly diagnosed peripheral T-cell lymphoma. Hematology.

[30] El-Azony A, Basha MAA, Almalki YE, Abdelmaksoud B, Hefzi N, Alnagar AA (2023). The prognostic value of bone marrow retention index and bone marrow-to-liver ratio of baseline 18F-FDG PET/CT in diffuse large B-cell lymphoma. Eur Radiol.

[31] Koh Y, Lee JM, Woo GU, Paeng JC, Youk J, Yoon SS (2019). FDG PET for evaluation of bone marrow status in T-cell lymphoma. Clin Nucl Med.

