# Supplementary material for: The utility of 18F-FDG PET/CT in assessing bone marrow involvement and prognosis in newly diagnosed diffuse large B-cell lymphoma
Source: Radiol Oncol. 2025 Dec 16;59(4):488–97. doi: 10.2478/raon-2025-0062 (PMC12707443; doi:10.2478/raon-2025-0062)
Supplement: Supplementary file 1 — Supplementary Material Details [file raon-2025-0062_sm.pdf]

The utility of ^{18}F-FDG PET/CT in assessing bone marrow involvement and prognosis in newly diagnosed diffuse large B-cell lymphoma

Chunyan Yang, Hong Liu, Furui Duan, Ximei Wang, Ping Li, Dalong Wang

doi: 10.2478/raon-2025-0062

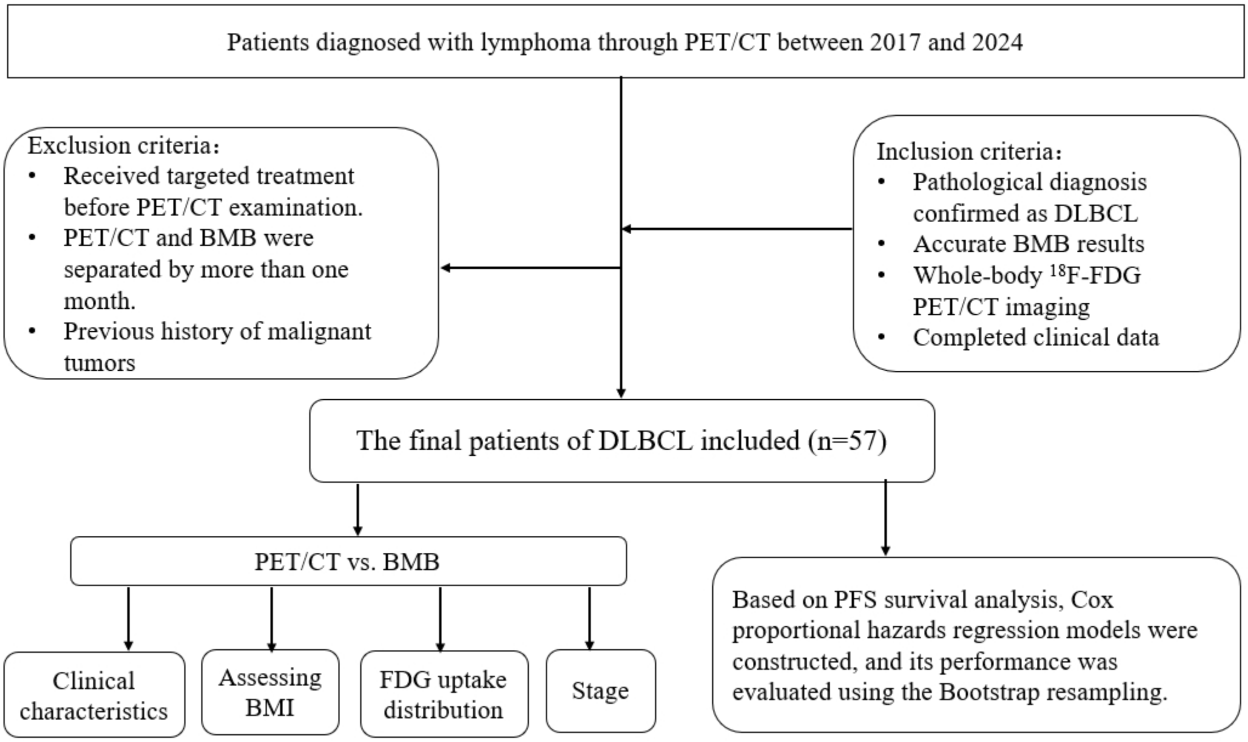

SUPPLEMENTARY FIGURE 1. Study flow diagram.

BMB = bone marrow biopsy; DLBCL = diffuse large B-cell lymphoma; PFS = progression-free survival

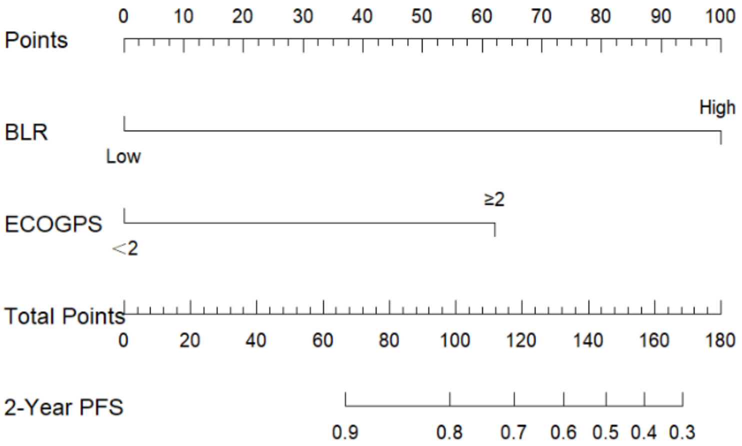

SUPPLEMENTARY FIGURE 2. Nomogram for the prediction of progression-free survival (PFS).

BLR = the ratio of the maximum standardized uptake values of bone marrow-to-liver; ECOGPS = Eastern Cooperative Oncology Group physical status score; PFS = progression-free survival

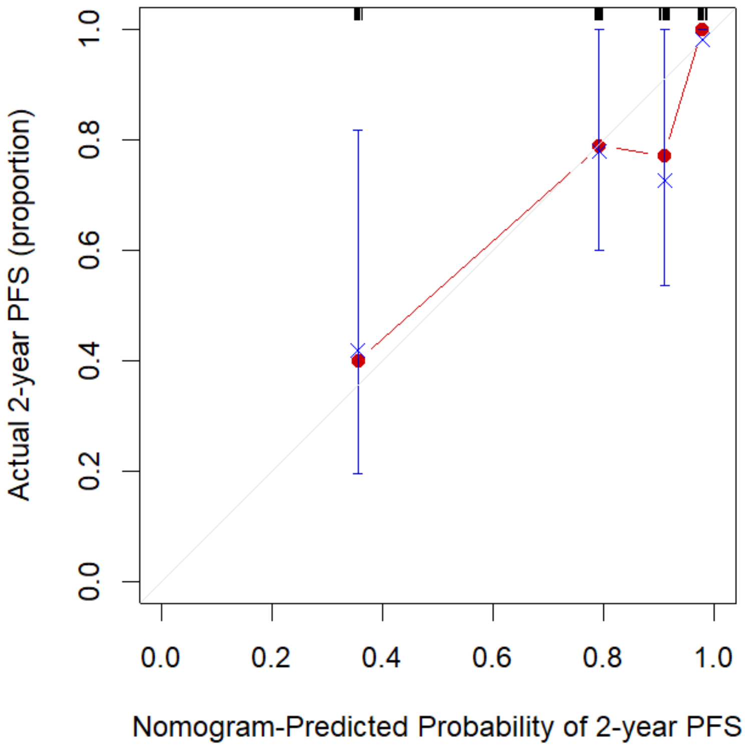

SUPPLEMENTARY FIGURE 3. Calibration curve.

PFS = progression-free survival
